# State of the Art in Fertility Preservation for Female Patients Prior to Oncologic Therapies

**DOI:** 10.3390/medicina56020089

**Published:** 2020-02-23

**Authors:** Călin Bogdan Chibelean, Răzvan-Cosmin Petca, Dan Cristian Radu, Aida Petca

**Affiliations:** 1 Department of Urology, George Emil Palade University of Medicine, Pharmacy, Science, and Technology of Targu-Mures, 540139 Targu-Mures, Romania; calinchibelean@yahoo.com; 2 Mureș County Hospital, 540136 Targu-Mures, Romania; 3 “Carol Davila” University of Medicine and Pharmacy, 050471 Bucharest, Romania; aidapetca@gmail.com; 4 Department of Urology, “Prof. Dr. Th. Burghele” Clinical Hospital, 050659 Bucharest, Romania; 5 Neolife Medical Center, 077190 Bucharest, Romania; dan.c.radu@gmail.com; 6 Department of Obstetrics and Gynecology, Elias University Emergency Hospital, 011461 Bucharest, Romania

**Keywords:** fertility preservation, cryo-preservation, vitrification, breast cancer

## Abstract

Quality of life improvement stands as one of the main goals of the medical sciences. Increasing cancer survival rates associated with better early detection and extended therapeutic options led to the specific modeling of patients’ choices, comprising aspects of reproductive life that correlated with the evolution of modern society, and requires better assessment. Of these, fertility preservation and ovarian function conservation for pre-menopause female oncologic patients pose a contemporary challenge due to procreation age advance in evolved societies and to the growing expectations regarding cancer treatment. Progress made in cell and tissue-freezing technologies brought hope and shed new light on the onco-fertility field. Additionally, crossing roads with general fertility and senescence studies proved highly beneficial due to the enlarged scope and better synergies and funding. We here strive to bring attention to this domain of care and to sensitize all medical specialties towards a more cohesive approach and to better communication among caregivers and patients.

## 1. Introduction

The female perspective on fertility after cancer treatment is, nowadays, an important issue. A 2012 study that analyses the information received from cancer survivors has found that there is a sex bias. Thus, men received more information regarding the influence of treatment on fertility 80% vs. 48%, and more men also received information about options to preserve fertility—68%, compared to 14% for women [1]. Statistics show that over 50% of men opted to cryopreserve sperm; only 2% of women undertook any means of fertility preservation [1]. The onco-fertility preservation necessity arises as 8% to 12% of all breast cancers occur before the age of 35 [2,3] and the tally rises to 15% for women 40 years old [4,5], in the backdrop of breast cancer being the most frequent of all cancers among women of childbearing age—affecting one-third of the young with cancer [6]. If we look at the breast cancer incidence in patients between the ages of 20 and 34 years, it stands at 1.9% of all newly diagnosed breast cancers and rises to 10.5% for breast cancers occurring in 35 and 44 year old women [7,8]. Breast cancer in young patients has special traits, characterized by specific oncogenic signaling pathways and associates a higher incidence of hormone receptor-negative, higher grade, and human EGF2 receptor-overexpressing tumors [9]. Advances in breast cancer early detection rates and treatment options have led to a five-year breast cancer survival rate of over 80% [10]. As greater survival rates are obtained, there is also a greater focus on achieving goals of motherhood and family completeness. Young Women’s Breast Cancer Study concluded that 50% of women younger than 40 years have concerns about future fertility and pregnancy options, following chemotherapy and radiotherapy [11]. There is also a psychological burden upon cancer survivors as a result of the fertility concern, and there are wide-spread studies to attest to the rising awareness of such instances [12].

A 2012 review of fertility demographics in USA showed an increase in the number of women giving birth after 30 years of age, with a peak for white women at 35 years [13]. The infertility risk of a woman in her teens is 0.2%, which will rise to 2% by her twenties, and reach 20% in her early thirties, which thus acknowledges only the number and quality of oocytes—by the time that most women will consider getting pregnant, they are already 20% infertile [14], resembling an infertility pandemic in developed countries. Adding to this is the increased incidence of cancer in young women, which will increase the cost of treatment and frequently implies infertility. Up to 6% of fertile age women are cancer survivors, and the incidence of cancer increases from about 1 in 10,000 shortly after birth to about 1 in 300 by mid-forties [15]. Depending on the source, ovarian failure characterizes 6.3% up to 12% of women that are childhood cancer survivors [16] and up to 50% of the patients that receive oncologic treatment at 40 years old will suffer early ovarian failure [17]. Most studies take account of the abrupt onset of menopause five years from chemotherapy, as evidence of ovarian failure, underestimating subtler manifestations such as subfertility and diminished ovarian reserve.

Chemo-therapeutic agents, known for deleterious effects, include alkylating agents that are considered high risk, such as Cyclophosphamide, Mechlorethamine, Chlorambucil, Busulfan, and Melphalan, whose active metabolites form DNA crosslinks leading to its function and synthesis arrest [18]. They produce DNA double-strand breakage, followed by P63 mediated apoptosis. Platinum-based compounds, Cisplatin and Carboplatin covalently binds DNA and forms intra and interstrand bonds that produce DNA strands breakage during replication. DNA transcription, synthesis, and function are, thus, inhibited. They are considered intermediate risk, though there was no demonstrated specific toxicity upon human primordial follicles [15,19]. Antimetabolites include Methotrexate, 5-fluorouracil, and Cytarabine and inhibit DNS synthesis and ARN purines and Thymidylate synthesis, being considered of low risk. Vinca alkaloids, Vincristine and Vinblastine inhibit tubulin polymerization, producing microtubules disruption during mitosis with mitosis arrest in metaphase and consecutive cellular death [3,18]. It is labeled as low risk. Anthracyclines, Daunorubicin, Bleomycin, Adriamycin (doxorubicin) inhibit DNA synthesis and function and inhibit Topoisomerase II leading to DNA breakage. They also form free oxygen radicals that also affect DNA synthesis and function by DNA breakage. Doxorubicin determines DNA double-strand breakage and human primordial follicles P63 mediated apoptosis. Except for Doxorubicin, which is considered of medium risk, they are low risk [18].

## 2. Current Fertility Preservation Methods

The 2018 American Society of Clinical Oncology (ASCO) recommendations paint a clear image of current onco-fertility focusing and emerging possibilities. While patients will first be taken aback by a cancer diagnosis, it is recommended that, early in the therapeutic process, discussions be initiated with the fertile patients concerning the risk of diminished fertility induced by specific treatment and about the options available for fertility preservation. Oocytes and embryo freezing, due to technological progress, are considered the standard of care and are widely available. Considering the conflicting evidence concerning GnRH agonist use to protect ovarian reserve during oncologic treatment [19], ASCO recommends only using GnRH agonists in young patients that are not fit for other methods. Embryo cryo-preserving is routinely used to preserve embryos that were not used for fresh embryo transfer after IVF. Oocyte freezing may be an adequate option for patients currently without a male partner, for those who do not want to use donor sperm or have ethical or religious objections regarding embryo cryopreservation. As of 2012, ASCO no longer considers oocyte cryopreservation experimental. As multiple ovarian stimulation protocols are available, there is no longer a need to delay ovarian stimulation depending on the menstrual cycle, favoring oocyte retrieval. For estrogen receptor-positive breast cancer patients that may be at risk due to the elevated estrogen levels in classic stimulation protocols, ASCO recommends aromatase inhibitors stimulation protocols as current studies do not show evidence of increased recurrence risk [20].

In this context of social pressure, and ultimately, financial stimulus, assisted reproduction technologies had a steep curve of evolution. In this process, IVF has greater visibility and striving for successful pregnancies produced more embryos. A limited number of the better-quality embryos are being selected for intrauterine embryo-transfer, while the remaining good quality embryos were frozen for cryopreservation. The resulting technological prowess led to increasing rates of embryo survival after freeze-thaw, and in turn, to a greater number of pregnancies, similar rates of pregnancies being obtained for fresh embryos and frozen ones. This highlighted the idea that all embryo-transfers should be realized with frozen embryos in a subsequent cycle; in favor of this judgment is the standing lower risk of ovarian hyperstimulation syndrome and a higher endometrial receptivity due to different gene expressions in stimulated versus unstimulated endometrium [21,22]. It seems that the cryo-preservation only strategy sustains a better chance for a viable pregnancy and lower abortion rates, and ovarian hyperstimulation has a 7% occurrence rate for fresh embryo pregnancies versus 1–3% for pregnancies with cryo-preserved embryos [23]. Frozen embryo pregnancies grew steadily, for example, from 28% in 2010 to 32% in 2011. Current trends contribute already by the discussed segmentation of IVF cycles, and pre-implantation genetic testing indicated the necessity by the advancing age of motherhood to the growing proportion of freeze-thaw cycles. Some countries like Switzerland, Finland, Holland, Iceland, and Sweden were fast to achieve 50% pregnancies from cryo-preserved embryos [24], mainly due to differences in embryo-transfer legislation and by the number limitation, which, in turn, increased the number of frozen embryos. As an answer to restrictive legislation like in Germany, Swiss, and Austria, or even interdiction as in Italy 2004–2009, oocyte-preserving techniques were fast emerging. Two types of cooling are used: slow freeze, which allows for cell dehydration, thus lowering ice formation and vitrification, creating a glass-like state by very fast cooling without ice formation. When cells are frozen very slow, excessive dehydration and shrinkage also lead to cellular death, and to avoid such an instance, cryo-protectants are typically used. Non-permeating cryo-protectants remain in the extracellular space, leading to the rising osmolality of the extracellular solution before freezing, and thus, preventing intracellular ice formation. The most used slow freezing cryoprotectant is a permeating agent, dimethyl sulfoxide—DMSO, and it was used as a cryo-protective agent for the first frozen-thaw human cleavage embryo transferred. DMSO displaces intracellular water; its cryo-protectant effect augments it with concentration but also increases cytotoxicity and clinical practice, as demonstrated by the toxic effect on patients [25]. The first successful mammalian embryo cryo-preservation was realized in 1972 by applying a cooling rate of ~1 °C/min up to −70 °C. This type of cooling is labeled equilibrium freezing, and the target is to maintain a sufficient intracellular dehydration rate to maintain intracellular water chemical potential in equilibrium with extracellular, partially frozen water. Mostly, ice formation occurs in the extracellular space with subsequent increases in cellular membrane osmotic pressure that accentuates after ice formation continues following nucleation due to the ice lattice excluding solvates that concentrate in the extracellular medium. Less permeable cell membranes rupture due to the abrupt rise in osmotic pressure if they cannot dehydrate fast enough. Intracellular ice formation may be lethal, and exposure to high concentrations of electrolytes may also cause cellular death [26]. Cryogenic injury is related to the cellular membrane permeability, and as a consequence, cells with higher membrane permeability have better survival rates at fast freezing rates, and cells with less permeable membranes need slower cooling. Vitrification was first used for mouse embryo cryopreservation, and became a viable alternative to conventional protocols, as it was shown to reduce cellular damage. During vitrification procedures, cells and tissues are exposed to cryo-protectants that dehydrate cells before commencing cooling. The most frequent method for embryo vitrification necessitates small specimen volumes to be exposed to super-fast cooling and warming rates. This technique was first used in human embryology by 1998 for cleavage stage embryos and then in 1999 for oocytes and pronuclear embryos. In the last 20 years, more vitrification methods were described, which involved a large array of cryo-preservers combinations such as Ethylene glycol (EG), DMSO, PROH (1,2-propanediol) and sucrose, Ficoll (polysaccharide solution), Trehalose, with varying parameters of dilution and equilibrium, support and cooling systems, storage and warming devices [27]. In 2005, Kuwayama M. et al. considered that the usual embryo and oocyte vitrification implied using a 15% solution of DMSO, 15% Ethylene Glycol, and 0.5 M sucrose in a very small volume ≤1 µL [28]. The fast cooling in vitrification is achieved by immersing the specimen in liquid nitrogen, and from this, two techniques are realized: open and closed vitrification. The majority of embryos and oocytes are vitrified by direct exposure to liquid nitrogen in the open system, favored because of its fast cooling and warming rates, increasing the method’s success [29]. The alternative is represented by the use of devices that mediate direct contact with liquid nitrogen-closed systems, presumably marred by lower cooling and thawing rates. It is currently considered that successful vitrification is closely dependent on successful cellular osmotic dehydration before cooling and on the warming rate than on the type and concentration of the cryo-preserving agent, so as to avoid water re-crystallization in the thawing cycle where very fast warming is a requisite. To highlight these conclusions, a recent study performed by a team that included Peter Mazur, one of the pioneers in the field, has demonstrated high survival rates after oocytes and embryos were vitrified without permeating cryoprotectants, and the thawing procedure was realized by ultra-fast warming by an infrared laser pulse [30]. Another study aimed to use a laser beam to dehydrate the blastocoel before vitrification and found substantial improvement in the clinical outcome by lowering the risk of ice recrystallization [31]. Vitrification is now considered the gold standard for oocyte and embryo cryopreservation [30]. Fast freezing protocols need a cell dehydration stage by cryo-protective agents to prevent ice formation, though the direct correlation between intracellular ice formation and cell death has yet to be well defined [26]. It seems that cell survival following cryo-preservation depends on the rate the cells are warmed during the thawing process, as cell damage does not occur during initial ice nucleation, but by another process during thawing and ice recrystallization [32] seems to be the main culprit. Among the first observations are the studies on organisms that naturally survive freezing and physiologically produce recrystallization inhibitors in large amounts [33]. Cryo-protectants based on carbohydrates represent an alternative to DMSO and have minimal or no toxicity; they act like glycerol, which is known to influence ice formation [34]. Ice recrystallization is inhibited by mono and poli-disaccharides, thus suggesting possible use in human cell cryopreservation. One study compared cellular viability after cryopreservation with mono and poli-disaccharides, and with the DMSO control. They found that the most powerful ice recrystallization inhibitors were 220 mM disaccharides solution, and the best viability was obtained with D-galactose 200 mM. It seems that the protective effect of D-galactose resides in its internalization, consequently lowering cellular osmotic stress [35]. Several studies used carbohydrates in the cryopreservation media, but did not strive to evaluate their efficiency, while in others, the success seemed dependent on the chemical structure or correlated carbohydrate efficiency with dehydration level of the milieu [36]. As recrystallization seems to be involved in cellular death derived from cryo-preservation [26,32], the intimate structural characteristic that is involved in recrystallization inhibition is not yet known. It is considered essential for the vitrification process’s success to limit to the minimum the amount of vitrification specimen to obtain a high rate of cooling and warming, thus preventing ice formation, and in this respect, oocytes are suitable freeze due to the low surface volume ratio that makes the cell membrane difficult to traverse for water and cryo-protectants [37]. Further, mature oocyte vitrification in metaphase meiosis (MII) may disrupt and deregulate the meiosis spindle, increasing the risk of chromosomal aberrations [38]. Unlike oocytes, embryos are more tolerant of freezing because membrane characteristics change after fertilization, favoring dehydration during cryo-preservation [39]. Embryo cryo-conservation led to the development of numerous devices that facilitate cooling and warming procedures, such as Cryoloop, nylon loop, Hemi-straw system, electron microscope plate, glass capillary, and Cryotop. Open Pulled Straw (OPS) was the first device specifically conceived for ultrafast vitrification, which was introduced by Vajta in 1998, and is still considered to be one of the best devices. The Cryotop is considered to be one of the most efficient vitrification methods both for oocytes and embryos, providing high rates of survival for both humans and animal models, and like other open cryo-preservation systems, directly contacts liquid nitrogen, increasing the risk of viral contamination [40]. Alternative methods were devised, such as micro-volume air-cooling (MVAC) that focused on preventing direct contact of the specimen with the liquid nitrogen [41].

There are patients for whom embryo cryo-preservation was not an option, and because oocyte cryo-preservation techniques had slower progress, ovarian tissue-slow-freeze represented the only fertility preservation method. Lately, oocyte cryo-preservation methods achieved good results, and the Practice Committee of the American Society for Reproductive Medicine (ASRM) reclassified oocyte cryopreservation technology as “nonexperimental” in 2013 [42]. While many oocyte preservation programs do not have long term data on oocyte preservation, especially for patients that received chemotherapy and radiotherapy treatment. Lastly, for increased chances of pregnancy, a few stimulation cycles could be necessary for oocyte preservation in light of evidence that using fresh oocytes only increases the chance of pregnancy by 5% [43].

## 3. New Techniques of Fertility Preservation

Ovarian tissue cryo-preservation may be an alternative to oocyte preservation, because for some patients’ ovarian tissue cryo-preservation, it may eliminate the need for the oncologic treatment delay needed for stimulation cycles for oocyte retrieval. There is hope that ovarian tissue transplant reinstates not only fertility, but also endocrine function. The slow freeze was compared with vitrification methods for the ovarian tissue [44] and some authors consider slow freezing a better method due to greater primordial follicle density and viability, less apoptotic cells and better morpho-functional aspects [44], while others did not find a significant difference in regards to these characteristics [45]. Shi et al. [46] showed that vitrification produces less DNA fragmentation regarding primordial follicles, and vitrification also produces superior results for the granulosa and stromal cells ultra-structure after vitrification. The large variety and the array of conflicting results stem from lack of standardization of the cryo-preservation protocols, the large spread of cryoprotective agents concentration, the variety of experimental animals, the varying implanting sites of the ovarian tissue, the different duration of observation protocols, and last but not least, the varying methods of success measurement. Clinical benefits of ovarian transplantation may be disputed, but, for sure, it benefits fundamental science on ovarian function, primordial follicle activation and development arrest as provided by studies such as those of Winkler et al., and more recently, Silber et al. and Hayashi et al. [47,48]. ASCO acknowledges that ovarian tissue cryopreservation and transplantation does not need ovarian stimulation and may be immediately performed. The added benefit resides in that it does not need sexual maturity, being the only method fit for children in the scope of fertility preservation and being able to restore the global ovarian function. As of 2018, ASCO considers ovarian tissue preservation still experimental, but keeps it open for evidence that can change this status [20]. Improving techniques led to an estimated 35%–40% live birth [49], since the first alive human baby, obtained as a result of ovarian cortex auto-transplant, was reported in 2004, and there were more than 100 live births worldwide; however, global reach of the procedure remains low, limiting further progress [49]. There are growing numbers of studies trying to discern how the best results can be obtained, by ovarian strip vitrification, or slow freezing, whole ovary versus ovarian strip, or the best place to insert the implant as one study concluded that implant location could significantly affect the results [50]. One group [51] compared whole ovary vitrification vs. slow freezing and concluded that the efficacy of whole ovaries cryopreservation by vitrification was higher than those by conventional freezing and rapid freezing, and that conventional freezing of ovarian cortical strips was more effective than cryopreservation of whole ovaries, independent of the way of whole ovary cryopreservation. Most of the live births after ovarian tissue cryopreservation, so far, have been achieved by slow controlled freezing, and only two teams achieved live births from ovarian tissue vitrification [52,53], while others still use the slow freezing method. Laboratories are striving to find the optimal concentration of cryo-protectants for the best results in ovarian tissue cryopreservation by vitrification. One of the pioneers of ovarian tissue vitrification, Silber S [53], reported that he and his team have used only ovarian tissue vitrification since 2008, after 11 years of slow freezing use. Vitrification may overcome the negative effects of freezing by inhibiting ice crystal formation, and also, vitrification has advantages related to its relatively lower cost, and does not require sophisticated freezing machines or ultra-specialized laboratory staff.

During the antenatal period, human ovaries lose to follicular atresia 80% of its germinal cells to roughly 500,000–1,000,000 at birth [54] and reaching puberty with only 300,000 to 500,000 oocytes; of these, just 400–500 will be selected for ovulation in the following 30–40 years and the rest will be extinguished. The 1% selected for ovulation is subjected to an FSH dependent process that leads to a dominant follicle that produces ovulation during the same cycle. It is considered that follicular activation in humans takes place in a wave pattern, taking effect even during pregnancy or contraceptive medication [54,55]. Follicular recruitment varies with age, from more than 1400 at the beginning of the third decade of life to less than 30 towards the end of the fifth decade and follicular destruction takes place in great numbers before or after follicular recruitment dependent or not on the menstrual cycle, with a decreasing rate during the lifetime, with more follicular loss occurring in young women [56]. The remaining ovarian follicle cohort thus declines in number throughout the lifetime, leading to reduced fertility in the fourth decade, irregular menses by the middle of the fifth decade, and menopause at around 50 years of age. This process remains in a mysterious equilibrium of reproductive aging and organismal aging as it is one of the most precocious aging phenomena in women, but new studies are bringing fresh hypotheses on how ovarian follicles are being activated. Some authors realized that following auto-transplant of ovary tissue after a very marked spike in AMH, the values stabilized at low levels. It has been described as a ‘burn out effect’, and there is still debate around the implicated mechanisms. One study concluded that the increased number of growing follicles versus resting follicles might be due to the downregulation of PTEN gene expression and subsequent augmentation of follicular recruitment [57]. A Japanese team led by Kawamura [58] recently proposed a premature ovarian insufficiency treatment by Hippo signaling dysregulation, realized by fragmenting ovarian tissue followed by Akt application and autografting. The serine/threonine kinase Akt (protein kinase B or PKB) has become a major focus of attention because of its critical role in regulating diverse cellular functions, including metabolism, growth, proliferation, survival, transcription and protein synthesis, while Hippo signaling is a conserved pathway regulating organ size by cell proliferation, apoptosis, and stem cell activity. It is thought that the disruption of the Hippo pathway contributes to cancer development. Other authors consider that follicle activation and ‘burn-out’ have an important contribution to post-implantation follicular depletion affecting ovarian tissue grafts [59]. SonerCelik et al. [60] research team’s findings regarding ovarian cryo-preservation and auto-transplantation demonstrate that expression of inhibitor proteins that control primordial follicle reserve decreases in cryopreserved ovaries after transplantation. The observation is consistent with the ovarian activity rush observed by others [61,62], and thus they debate the recommendation of follicular activation prior (in vitro activation IVA) to transplantation [58,63]. The longevity of the transplanted ovarian tissue varies widely and may depend on the age of the woman at cryo-preservation [64], some blaming the revascularization rate after transplantation as an important issue [65]; however, long functioning viability of more than ten years has been reported [66]. This success has promoted ovary cryo-preservation for potential use in severe genetic conditions with a risk of primary ovarian insufficiency like sickle cell anemia, thalassemia, Turner syndrome, and galactosemia [67]. A study performed mathematical modeling after losing 50% of the ovarian reserve after mono ovariectomy and concluded that the maintenance of ovarian function suggests an extra-ovarian, probably an age-dependent regulation agent of reproductive decline [68]. A review of cases comprising women with unilateral oophorectomy concluded that the menopause age was lowered by only 1.8 years [69]. By the same logic, it has been suggested that extracting ovarian strips early in life would not substantially affect menopause age, but by cryo-preservation, the ovarian tissue would later be re-implanted, thus conveniently delaying menopause [70]. A proposed mechanism for this ovarian function refers to the downregulation of follicular activation as the follicle pool is diminishing [71]. Another method described for fertility augmentation refers to autologous stem cell ovarian transplantation with, seemingly, relative success [72]. Tilly et al. have long provoked the fertility dogma that women dispose from birth of a fixed, limited number of follicles, by affirming the existence of oogonial stem cells that can be activated, thus jumpstarting fertility [73]. There are reports that sphingosine-1-phosphate has cytoprotective functions in human ovaries, two studies showing that S1P reduces primordial follicle loss in human ovarian tissue xenografted in mice and exposed to cyclophosphamide as an in-vivo model of chemotherapy-induced ovarian damage [74,75]. Another study suggested the use of Sphingosine-1-phosphate to reduce the follicular atresia occurring during the freeze-thawing procedure [76].

As an alternative to ovarian tissue transplants, probing the hypotheses that limited success with ovarian tissue strips is due to the limited and late graft vascularization, there were animal and human trials with whole ovary transplants with limited success [77]. Some limitations were linked to the sheer mass of the ovary (sometimes animal trial included bovine ovaries) posing difficulties in the freezing process, being slow or exhibiting vitrification even after cannulation of the main vessels with cryo-protectants and likely experiencing ice recrystallization in the thawing [78]. Another obstacle is presented by the reperfusion lesions for the prevention, for which some authors tried edaravone, as it is supposed to relieve oxidative stress [79]. An observed supplemental difficulty results from the extensive dissection needed for extraction, but mostly for the ovarian implant, especially venous anastomosis due to the thin venous walls even for experienced teams like M Brännström’s that is pioneering uterus transplantation [80].

Owing to the dispute that entangles the use of GnRH agonists [19], alternatives are looked for with the scope to protect ovarian function during chemotherapy. One example of such a protecting agent is represented by Sphingosine-1-phosphate [74,75], others examined co-administration of imatinib, (a 2-phenyl amino pyrimidine derivative that inhibits activity of the tyrosine kinase domains of c-Abl, c-Kit) and platelet-derived growth factor receptor, as they have been reported to attenuate follicle depletion in mice caused by cytotoxic treatments [81], although other studies failed to evidence the protective effect [82]. Anti-Müllerian hormone (AMH) represents another example of hopeful agents to be used in fertility preservation. AMH is part of the transforming growth factor (TGF)-beta family, with a central role in the control of sexual differentiation and follicular genesis, and while serum AMH levels have long been used in reproductive medicine as an indicator of ovarian reserve, it is now investigated as a protective agent [83].

The advent of new technologies brought new fields of research, and while ovarian strip auto-transplant harbors hope for both fertility preservation after oncologic therapy and menopause delay, studies searching for artificial support or even fully artificial ovaries are in full stride. A team is proposing a bioprosthetic ovary that was assembled using 3D printed microporous scaffolds in order to restore ovarian function [84]. Additionally, this is not a singular example since the oncofertility field is evolving; meanwhile, eager bioengineers have sought to create artificial ovaries with biomaterials and isolated follicles [85].

## 4. Conclusions

Societal pressures pushed forward the long and successful experience with ART due to the increasing age of childbearing, which, in turn, exposed women intending to procreate to a higher risk of malign conditions. This is coupled with the better chance and longer disease-free survival by novel chemotherapy schemes that produce a growing population of women trying to conceive at increasing ages, often after oncologic treatment. Oncofertility studies crosslink with female aging studies and general fertility. There are promising technologies from oogonial stem cells activation, artificial scaffold bioprosthetic ovaries, and proactive ovarian tissue extraction for future use that could push female fertility farther away into what is now senescence.
